# Supplementary material for: Social Inequities in the Impact of COVID-19 Lockdown Measures on the Mental Health of a Large Sample of the Colombian Population (PSY-COVID Study)
Source: J Clin Med. 2021 Nov 15;10(22):5297. doi: 10.3390/jcm10225297 (PMC8619612; doi:10.3390/jcm10225297)
Supplement: Supplementary file 1 [file jcm-10-05297-s001.zip › jcm-1395358-supplementary.pdf]

Table S1. Mental health outcomes for each socio-demographic characteristic.

Variables	Depression (PHQ-2) (range: 0-6) M (SD)	Anxiety (GAD-2) (range: 0-6) M (SD)	Somatization (SSQ-5) (range: 0-15) M (SD)
Gender			
Female	2.34 (1.60)	2.08 (1.68)	3.92 (2.86)
Male	2.09 (1.63)	1.77 (1.63)	2.77 (2.57)
Non-binary	3.15 (1.99)	2.61 (1.94)	4.52 (3.77)
Age group			
Young adults (18-29 years)	2.78 (1.64)	2.29 (1.77)	4.29 (3.03)
Middle aged adults (30-59 years)	1.95 (1.49)	1.82 (1.57)	3.26 (2.59)
Older adults (≥ 60 years)	1.48 (1.37)	1.50 (1.42)	2.06 (2.14)
Income level			
Low	2.69 (1.70)	2.27 (1.80)	3.98 (3.07)
Medium	2.15 (1.54)	1.92 (1.61)	3.57 (2.75)
High	1.88 (1.54)	1.76 (1.55)	3.05 (2.51)
Work status			
Student	2.69 (1.68)	2.26 (1.77)	4.15 (2.99)
Informal workers	2.13 (1.56)	1.93 (1.67)	3.36 (2.75)
Formal workers	2.18 (1.58)	1.95 (1.63)	3.60 (2.78)
Unpaid workers	2.30 (1.59)	2.04 (1.70)	3.43 (2.78)
Unemployed	2.25 (1.59)	1.98 (1.67)	3.58 (2.84)
Retired	1.84 (1.50)	1.68 (1.53)	2.63 (2.50)
Education level			
No studies	2.11 (1.84)	2.61 (2.28)	5.33 (4.69)
Primary	2.25 (1.74)	2.06 (1.80)	3.10 (3.01)
Secondary	2.42 (1.71)	2.14 (1.82)	3.14 (2.86)
University	2.27 (1.60)	1.99 (1.65)	3.70 (2.82)
Ethnic group			
Gypsy	2.19 (1.44)	1.88 (1.53)	3.00 (2.13)
Afrodescendant	2.35 (1.63)	2.02 (1.70)	3.40 (2.90)
Indigenous	2.33 (1.70)	2.08 (1.76)	3.45 (2.86)
None of the above	2.27 (1.61)	2.00 (1.67)	3.66 (2.83)
Area of residence			
Urban	2.29 (1.61)	2.02 (1.68)	3.67 (2.83)
Rural	2.14 (1.56)	1.87 (1.61)	3.27 (2.82)
Region			
Amazon	2.34 (1.70)	2.00 (1.66)	3.89 (3.10)
Andean	2.20 (1.56)	1.97 (1.63)	3.66 (2.81)
Caribbean	2.22 (1.58)	1.95 (1.66)	3.66 (2.98)
Orinoco	2.32 (1.68)	2.07 (1.79)	3.58 (3.00)
Pacific	2.40 (1.67)	2.06 (1.72)	3.61 (2.82)

Note. M = Mean, SD = Standard deviation.
